# Balancing excitation and inhibition in the autistic brain

**DOI:** 10.7554/eLife.60584

**Published:** 2020-08-04

**Authors:** Charlotte M Pretzsch, Dorothea L Floris

**Affiliations:** 1 Department of Forensic and Neurodevelopmental Sciences, Kings' College LondonLondonUnited Kingdom; 2 Donders Centre for Cognitive NeuroimagingNijmegenNetherlands

**Keywords:** autism, Hurst exponent, sex/gender, excitation, inhibition, fMRI, Human, Mouse

## Abstract

A metric called the Hurst exponent could be a useful biomarker for studies exploring brain differences between men and women with autism spectrum disorder.

**Related research article** Trakoshis S, Martínez-Cañada P, Rocchi F, Canella C, You W, Chakrabarti B, Ruigrok AN, Bullmore ET, Suckling J, Markicevic M, Zerbi V, MRC AIMS Consortium, Baron-Cohen S, Gozzi A, Lai MC, Panzeri S, Lombardo MV. 2020. Intrinsic excitation-inhibition imbalance affects medial prefrontal cortex differently in autistic men versus women. *eLife* **9**:e55684. doi: 10.7554/eLife.55684

Balance and stability are important for the human body. This is clearly true for some physical tasks, such as walking or running without falling over, but it is also true inside the brain. Neurons receive both excitatory and inhibitory inputs, and maintaining a balance between the two – that is, maintaining what is known as the excitation-inhibition or E:I balance – is crucial for the brain to work properly (Yizhar et al., 2011).

Disruptions to the E:I balance, such as increased levels of excitatory inputs, have been linked to autism and a number of other neurological conditions, and can affect brain function and social behavior (Rubenstein and Merzenich, 2003). It has also been shown that the E:I balance can depend on sex, especially in regions of the brain that support social behavior, such as the ventromedial prefrontal cortex (Lai et al., 2019). However, research into the relationships between autism, sex and social behavior have been hampered by a lack of non-invasive tools. Now, in eLife, Michael Lombardo (Istituto Italiano di Tecnologia and University of Cambridge) and colleagues – including Stavros Trakoshis and Pablo Martínez-Cañada as joint first authors – report how it may be possible to measure the E:I balance without causing damage to the brain (Trakoshis et al., 2020).

The researchers – who are based in Italy, Cyprus, South Korea, the United Kingdom, Switzerland, and Canada – first created a computational model of the brain and used data from this model to calculate a metric called the Hurst exponent. This metric is a measure of the long-term memory of a time series of data. The model was then tweaked to simulate the effect of a ligand called CNO (which is short for clozapine-N-oxide) on two receptors hM3Dq and hM4Di, which are synthetic proteins designed to bind synthetic drugs. When CNO binds to hM3Dq it increases excitation, and when it binds to hM4Di it reduces both excitation and inhibition. Trakoshis et al. found that the change in the E:I balance caused by CNO also changed the Hurst exponent: specifically, an increase in excitation decreased the Hurst exponent (Figure 1).

**Figure 1.**
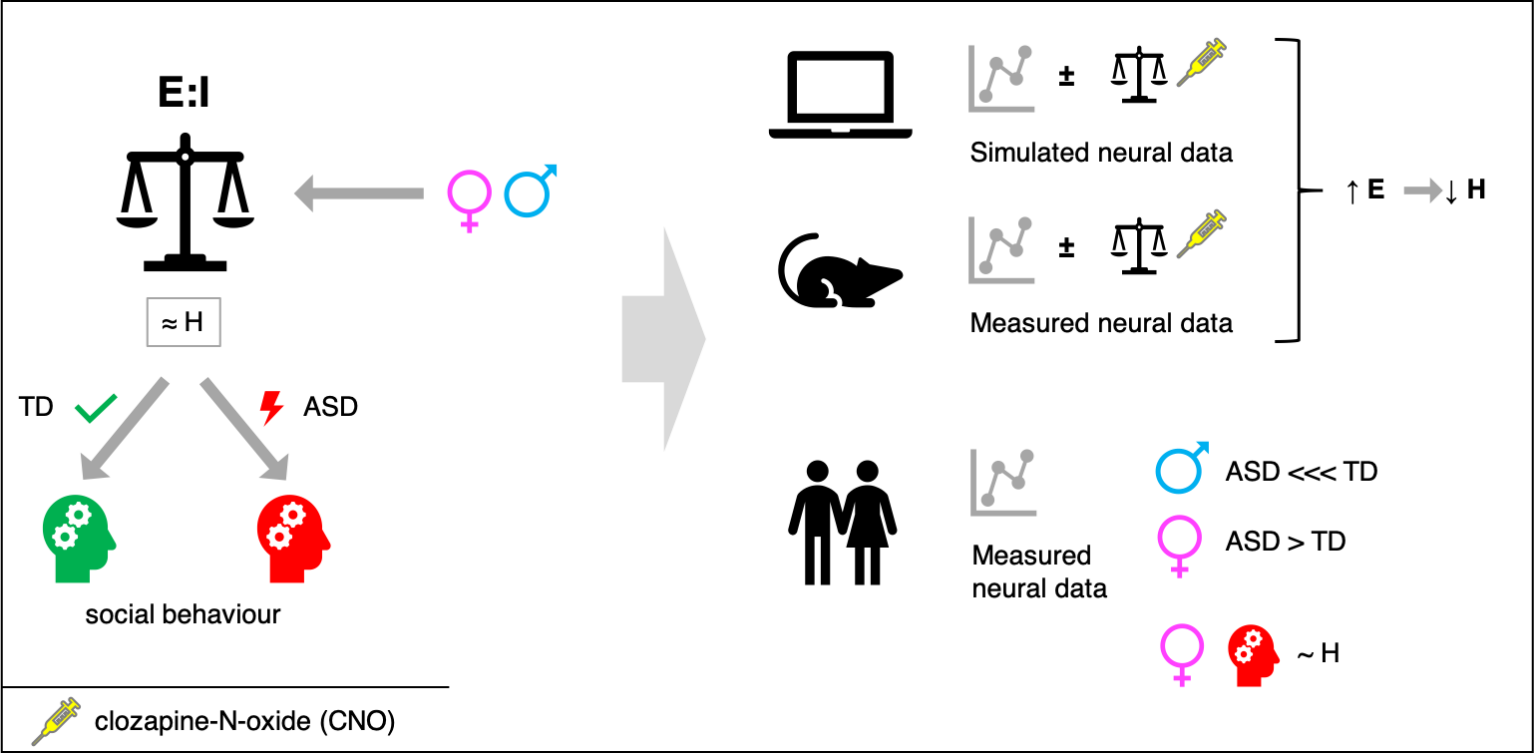
Maintaining a balance of excitatory and inhibitory input in neurons. An imbalance between excitatory and inhibitory inputs to neurons in certain regions of the brain has been linked to neuropsychiatric conditions such as autism spectrum disorder (ASD). However, it is not possible to measure such imbalances directly, so it would be useful to have a proxy marker. Based on computer simulations and experiments on genetically manipulated mice (top), Trakoshis et al. found that a metric called the Hurst exponent, which can be determined using non-invasive electrical and magnetic measurements, can provide information on imbalances between excitatory and inhibitory input. In particular, an increase in excitation (E) led to a decrease in the Hurst exponent (H). Experiments on autistic and typically developing (TD) men and women show that: i) the value of H for men with autism is much lower than the value for TD men; ii) the value of H for women with autism is higher than the value for TD women; iii) in women with autism, the value of H further correlated with social behavior.

Second, the researchers tested if they could replicate these computational results in mice that had undergone chemogenetic manipulation to express hM3Dq and hM4Di. This involved measuring electrical activity in the prefrontal cortex of the mice before, during and after the administration of CNO, and then computing the Hurst exponent. They found that the in vivo results in mice confirmed the predictions of their computational model. Specifically, a drug-induced increase in excitation led to a decrease in the Hurst exponent.

Third, Trakoshis et al. wanted to find out why E:I processes may differ between males and females. They found that genes associated with autism that also affect the E:I balance (especially excitation) overlapped with genes that are sensitive to male-specific hormones. A comparison of these results with a gene expression map called the Allan Human Brain Atlas (Hawrylycz et al., 2012) further revealed that these genes are expressed in many regions of the brain, including the ventromedial prefrontal cortex.

To investigate how the E:I balance may differ between autistic men and women, and how those differences may relate to social behavior, Trakoshis et al. collected neural time series data and computed the Hurst exponent in individuals with and without autism (who were similar in age and IQ). In the ventromedial prefrontal cortex, the Hurst exponent of individuals with autism was lower than that of typically developing individuals, with the difference between the two being larger in males than in females. Moreover, in women with autism, the Hurst exponent was linked with social behavior: a higher (i.e., more typical) value was accompanied by a better ability to 'socially camouflage' – that is, the ability to compensate for social-communicative difficulties.

Taken together, these results suggest that the Hurst exponent may be a useful biomarker to examine the E:I balance and its relationship with sex and social behavior across the autism spectrum. More research is needed to investigate if shifting the E:I balance in the ventromedial prefrontal cortex could alter social behavior in people with autism. In the future, this approach could be extended into other conditions linked to E:I balance and sex-differences, such as attention deficit hyperactivity disorder.
